# Non-operative Management of an Isolated Blunt Traumatic Retrohepatic Inferior Vena Cava Injury

**DOI:** 10.7759/cureus.36746

**Published:** 2023-03-27

**Authors:** Frederique Pinto, Reginald Alouidor, Sheina Theodore

**Affiliations:** 1 Division of Trauma, Critical Care and Acute Care Surgery, Baystate Medical Center, Springfield, USA; 2 Department of Acute Care Surgery and Trauma, Boston Medical Center, Boston, USA

**Keywords:** inferior vena cava injury, hepatic trauma, blunt trauma, non-operative management, traumatic inferior vena cava injury, blunt abdominal trauma

## Abstract

Traumatic inferior vena cava (IVC) injuries are often fatal. Blunt IVC injuries are encountered less often. Conservative management, albeit an option, is not often discussed in the literature. This report explores the non-operative management of a 52-year-old female unrestrained driver who presented with a blunt retrohepatic IVC injury identified on a computed tomography (CT) scan that revealed IVC disruption with extravasation of contrast. Here, we discuss the nonoperative management of the patient and review the literature concerning IVC anatomy, traumatic injuries, and management. We conclude that a hemodynamically stable patient with an isolated blunt traumatic IVC injury can be managed non-operatively.

## Introduction

Traumatic inferior vena cava (IVC) injuries are rare and often fatal, with a pre-hospital mortality rate as high as 30-50% [1]. While these are predominantly the result of penetrating traumatic injury, they only represent <5% of penetrating trauma and <1% of blunt traumatic injuries [2]. Blunt traumatic vena caval injuries are generally associated with significant injury mechanisms and are encountered in multi-system trauma patients, where management tends to require numerous surgeries and a prolonged hospital stay. While these injuries are typically managed surgically, the operative access to a high-flow, low-pressure venous system makes this particularly challenging [2], and there is little data to guide selective non-operative management in the stable patient. Here, we present a rare case of an isolated blunt traumatic retrohepatic caval injury that was managed non-operatively.

## Case presentation

**Figure 1:**
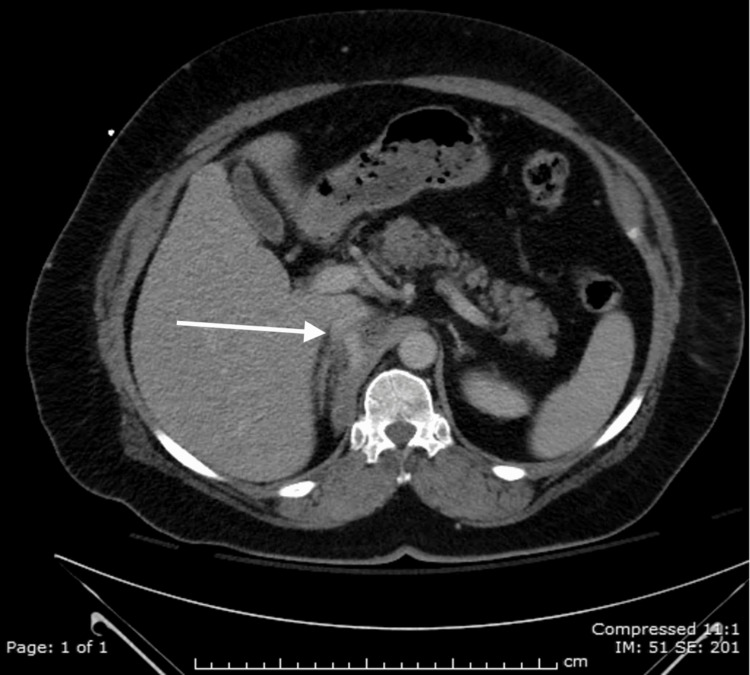
Axial view of the retrohepatic inferior vena cava disruption with pericaval hematoma (white arrow).

**Figure 2:**
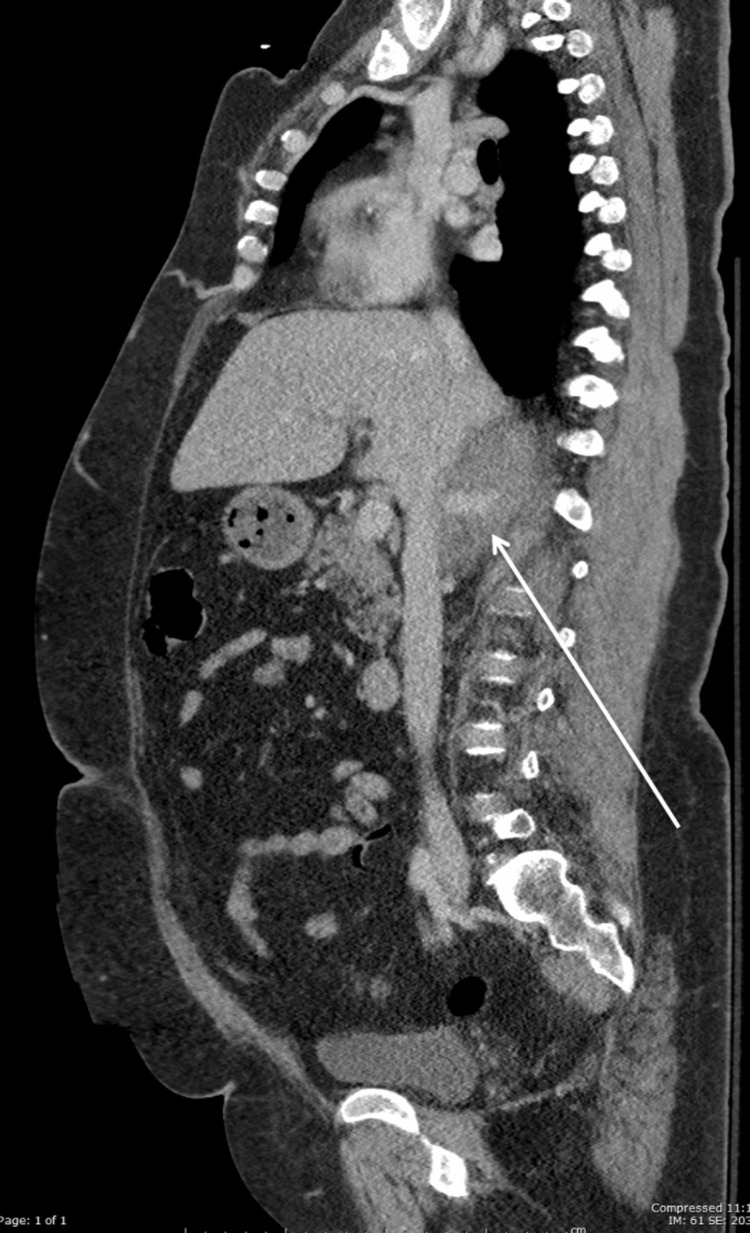
Sagittal view of retrohepatic IVC disruption with contrast extravasation (white arrow). IVC: inferior vena cava.

A 52-year-old female was an unrestrained driver involved in a single motor vehicle crash into a guardrail. At the scene, the patient was found to be intoxicated with a Glasgow Coma Score (GCS) of 13 by pre-hospital personnel. She was transported via ambulance to our level 1 trauma center. On arrival, her GCS was 14, she was hypertensive with a systolic blood pressure (SBP) of 180 mmHg and other vital signs were within normal limits. She appeared to be intoxicated and was intermittently agitated and combative. The secondary survey revealed contusions of the left chest wall and anterior lower extremities bilaterally. The abdominal exam was benign: soft, non-distended, and non-tender. A chest x-ray was obtained in the trauma bay and revealed no acute pathology. Secondary to persistent complaints of back pain and an altered mental status, a computed tomography (CT) scan of the chest, abdomen, and pelvis was performed to assess the thoracic and lumbar spines. Laboratory studies were significant for a hematocrit of 41.7. The IV contrast CT scan of the abdomen revealed an isolated retrohepatic inferior vena cava (RIVC) injury with a contrast-enhancing blush, consistent with IVC disruption and active venous bleeding (Figures 1, 2).

Due to her stable clinical exam, she was admitted to the surgical intensive care unit (SICU) for observation and close hemodynamic monitoring. Her GCS improved to 15. Repeat serial hematocrits showed a decline to 36.9 at 36 h post-admission but remained stable thereafter (Table 1).

**Table 1:** Hematocrit trend.

Time	Hematocrit
Arrival	41.7
12 h post-event	37.5
24 h post-event	36.2
36 h post-event	36.9

A follow-up CT abdomen and pelvis performed on hospital day two revealed minimal expansion of the retrohepatic hematoma consistent with tamponade with no further evidence of extravasation. She was discharged on hospital day two. On the day of discharge, she tolerated a regular diet, was hemodynamically stable, and the abdominal exam remained benign (soft, non-distended, and non-tender).

## Discussion

Blunt inferior vena cava (IVC) injuries are frequently lethal. Fifty percent of these patients die in the field, and the mortality for those who survive to the hospital is 20-57% [1,3,4]. Although the majority of IVC injuries result from penetrating trauma (85-95%), up to 14% result from blunt trauma [5,6]​​​. Isolated IVC injuries secondary to blunt trauma are a rarity, with few case reports in the literature [3,6]. The IVC begins at the union of the common iliac vessels, ascends to the right of the vertebrae, has many contributory vessels, and crosses the diaphragm to join the right atrium. The abdominal IVC is anatomically divided into five segments: retrohepatic, suprarenal/subhepatic, perirenal, infrarenal, and the inferior bifurcation region. We report a rare case of blunt trauma resulting in an isolated retrohepatic inferior vena cava (RIVC) injury treated non-operatively. Blunt retrohepatic IVC injuries generally stem from shear forces associated with deceleration because the unique anatomy of this region places the IVC in a free-floating position relative to the IVC portions located immediately superior and inferior to the retrohepatic region [1]. RIVC injuries can be contained spontaneously by anatomical tamponade between the diaphragm and liver posteriorly and anteriorly, respectively, and the hepatic suspensory ligaments surrounding the IVC [1]. The lower pressure of the venous system, as compared to the arterial system, allows the stabilization of the clot. This is further enhanced by ensuring low venous pressure with conservative use of intravenous fluid and close central venous pressure monitoring [7]. In our case, the patient arrived in the trauma bay with no sign of shock indicative of spontaneous tamponade of the IVC injury [1]. The diagnosis of the IVC injury was made after imaging for lower back pain. The diagnosis of the IVC injury on CT scan with IV contrast includes retroperitoneal hematoma located paracavally, irregular vena caval contour, and extravasation of blood from the vena cava [5]; all these findings were present on the CT scan in this case. 

This patient had extravasation of contrast in the retroperitoneum, an area of high vascularity. In general, this is an ominous sign. In this hemodynamically stable patient with the inherent risks of surgical exploration of a RIVC injury, the trauma team, after consultation with vascular surgery, elected to manage the patient conservatively with surgical intensive care unit (SICU) admission for continuous hemodynamic monitoring. Surgical intervention is not always required in the presence of spontaneous tamponade; success has been noted with conservative management and observation in the past [7]​​​​​​. Some 10-40% of patients who undergo surgery inadvertently release an uncontrolled bleed secondary to the release of an anatomical tamponade [1]​​​​​​.

We limited imaging studies to CT scans with IV contrast. A repeat abdominal CT was performed and timed to look specifically at the venous phase. As the patient was clinically stable with vital signs and hematocrit within normal limits in conjunction with a benign abdominal exam, one could argue that a repeat CT resulted in an increased risk of contrast-induced nephropathy and increased cost with limited to no benefit to the patient. Other potential imaging modalities include a cavogram, which we elected not to perform given concerns that instrumentation of the IVC or a high-pressure injection could potentially dislodge a clot and lead to bleeding complications. There was no clear benefit of this procedure for this patient.

Non-operative interventional radiology procedures can be an option for the control of RIVC injuries. There are case reports in the literature about using balloon catheter occlusion to control an inferior vena cava injury [8]. Endovascular surgery can also be used to occlude the injury and may be an option, but this requires proper facilities, an experienced interventional team, and the availability of an assortment of devices [9]​​​​​​​. If this patient did require surgery, our institution has a hybrid interventional radiology/surgery suite where the RIVC could be controlled by venous endovascular techniques with balloon occlusion proximally and distally to gain vascular control or by temporary placement of an endovascular graft before an open surgical exploration. Balloon occlusion of the RIVC would invariably result in a significant decrease in the cardiac preload and potentially induce shock. Given the location of the injury, an endovascular graft would be feasible. However, with the risk of occluding the subhepatic veins and potentially the renal vessels, this would be the equivalent of the placement of an atriocaval Shrock shunt that is often quoted in the literature but almost universally fatal [2]. Both of these options can be entertained in the setting of emergency surgery in the unstable patient, as patients who die from IVC injury typically succumb to intraoperative exsanguination [8]​​​​​​​. As this patient was stable, we agreed that the risks of pursuing endovascular control outweighed the benefits.

The factors that predict mortality in patients with traumatic IVC injuries were studied in a respective review performed by Huerta et al. The Glasgow Coma Score (GCS) is an independent predictor of survival for patients with blunt IVC injury, with a decreased GCS as a poor prognostic indicator. Other poor prognostic predictors include low systolic blood pressure (SBP), high injury severity score (ISS), and emergent thoracotomy [4]​​​​​​​. Given the patient had an initial GCS of 13, which improved to 15, an isolated retrohepatic IVC injury and hypertension, a favorable prognosis was expected.

## Conclusions

RIVC injuries are rare and typically present in unstable patients with multiple injuries. These patients often require emergency surgery and tend to have poor outcomes. More recently, endovascular techniques have been successfully used to manage these patients. Our case illustrates that non-operative management of a hemodynamically stable patient with an isolated blunt retrohepatic inferior vena caval injury using serial hematocrit measurements and repeat CT with IV contrast (focusing on the venous phase) is an option in select cases.
